# Exclusive Human Milk Diet for Extremely Premature Infants: A Novel Fortification Strategy That Enhances the Bioactive Properties of Fresh, Frozen, and Pasteurized Milk Specimens

**DOI:** 10.1089/bfm.2022.0254

**Published:** 2023-04-14

**Authors:** Roy K. Philip, Ehab Romeih, Elizabeth Bailie, Mandy Daly, Kieran D. McGourty, Andreas M. Grabrucker, Colum P. Dunne, Gavin Walker

**Affiliations:** ^1^ Division of Neonatology, Department of Pediatrics, University Maternity Hospital Limerick (UMHL), Limerick, Ireland.; ^2^ University of Limerick School of Medicine, Limerick, Ireland.; ^3^ Department of Chemical Sciences and Bernal Institute, University of Limerick, Limerick, Ireland.; ^4^ Milk Bank, Western Health & Social Care Trust, Enniskillen, Northern Ireland.; ^5^ Advocacy and Policymaking, Irish Neonatal Health Alliance (INHA), Wicklow, Ireland.; ^6^ Bernal Institute, University of Limerick, Limerick, Ireland.; ^7^ Health Research Institute (HRI), University of Limerick, Limerick, Ireland.; ^8^ Department of Biological Sciences, University of Limerick, Limerick, Ireland.; ^9^ Center for Interventions in Infection, Inflammation, and Immunity (4i), University of Limerick School of Medicine, Limerick, Ireland.

**Keywords:** exclusive human milk diet, human milk-derived fortifier, donor human milk, breastfeeding, pasteurization, necrotizing enterocolitis, donor milk bank

## Abstract

**Background:**

Human milk (HM) fortification has been recommended for the nutritional optimization of very low–birthweight infants. This study analyzed the bioactive components of HM and evaluated fortification choices that could accentuate or attenuate the concentration of such components, with special reference to human milk-derived fortifier (HMDF) offered to extremely premature infants as an exclusive human milk diet.

**Materials and Methods:**

An observational feasibility study analyzed the biochemical and immunochemical characteristics of mothers' own milk (MOM), both fresh and frozen, and pasteurized banked donor human milk (DHM), each supplemented with either HMDF or cow's milk-derived fortifier (CMDF). Gestation-specific specimens were analyzed for macronutrients, pH, total solids, antioxidant activity (AA), *α*-lactalbumin, lactoferrin, lysozyme, and α- and *β*-caseins. Data were analyzed for variance applying general linear model and Tukey's test for pairwise comparison.

**Results:**

DHM exhibited significantly lower (*p* < 0.05) lactoferrin and α-lactalbumin concentrations than fresh and frozen MOM. HMDF reinstated lactoferrin and α-lactalbumin and exhibited higher protein, fat, and total solids (*p* < 0.05) in comparison to unfortified and CMDF-supplemented specimens. HMDF had the highest (*p* < 0.05) AA, suggesting the potential capability of HMDF to enhance oxidative scavenging.

**Conclusion:**

DHM, compared with MOM, has reduced bioactive properties, and CMDF conferred the least additional bioactive components. Reinstatement and further enhancement of bioactivity, which has been attenuated through pasteurization of DHM, is demonstrated through HMDF supplementation. Freshly expressed MOM fortified with HMDF and given *early*, *enterally*, and *exclusively (3E)* appears an optimal nutritional choice for extremely premature infants.

## Introduction

One in 10 infants globally and 8.7% of births in Europe are premature.^1^ While breast milk (BM) offers the natural enteral feeding choice for preterm infants, optimal gestation-specific nutrition of the most immature neonates warrants nutritional enrichment or fortification of human milk (HM), intending to approximate fetal levels of tissue accretion and growth.^2–4^ Even though the guiding tenets of preterm nutrition emphasize early and exclusive HM uptake, adding exogenous cow's milk-derived fortifier (CMDF) remained the standard practice for decades.^5^ Recent recognition of nutritional limitations and risks posed by such bovine-origin protein supplementation stimulated a novel enrichment using human milk-derived fortifier (HMDF).^6–9^ An exclusive human milk diet (EHMD) for very preterm (VPT, <32 weeks of gestational age) and very low–birthweight (VLBW, <1,500 g birth weight) infant comprises mother's own milk (MOM) or pasteurized banked donor human milk (DHM) fortified with HMDF.^9^

Proposed benefits of EHMD in neonatal intensive care unit (NICU) include the reduction of necrotizing enterocolitis (NEC), late-onset neonatal sepsis, bronchopulmonary dysplasia, retinopathy of prematurity, total parenteral nutrition usage, and length of stay.^9–13^

It has not yet been determined with certainty which immunologic or bioactive substances in the unique HM matrix impart the specified protective properties. Irrespective of this uncertainty, in situations where MOM is unavailable or insufficient for VLBW infants, the American Academy of Pediatrics and World Health Organization (WHO) recommend DHM as the next best proxy enteral nutritional choice and as a bridge to establish MOM.^2–4^ It is estimated that over 800,000 infants worldwide receive DHM annually, and multiple studies underscore short- and long-term benefits of DHM over bovine-origin preterm formula (PTF).^14–16^

DHM composition and properties are subject to the complexity of processing involving storage, freezing, pasteurization, thawing, and homogenization, resulting in quantitative and qualitative attenuation of bioactive proteins and immunomodulatory components.^17–20^ In this context, it is reasonable to suggest that even when fortifiers are added to freshly expressed mother's own milk (FreMOM), frozen mother's own milk (FroMOM), preterm donor human milk (PTDHM), and full-term donor human milk (FTDHM), all of which are variably used during the course of the NICU stay of VPT and VLBW infants; significant nutritional, biochemical, immunological, and microbiological variations exist potentially influencing the clinical outcomes.^9,13,21^

As the Irish breastfeeding rate is considered one of the lowest globally, optimizing the uptake of HM for preterm infants poses additional clinical and cultural challenges.^22^ Our objective was to analyze the selected biochemical and bioactive characteristics of enteral feeding choices used for extremely preterm (<28 weeks gestation) and extremely low-birthweight (ELBW, <1,000 g birth weight) infants at the first NICU in Ireland that established an EHMD for the most vulnerable premature infants.^23^ In doing so, we believe that the analysis will provide new insight into the differences in the bioactivity of MOM and DHM and evaluate the role of HMDF to accentuate or reinstate the concentrations of such fractions.

## Methods

An observational feasibility study of biochemical and immunochemical analysis of HM specimens was conducted, with and without various fortification regimens. An analysis of macronutrients, pH, total solids, antioxidant activity (AA), *α*-lactalbumin (*α*-LA), lactoferrin (LF), lysozyme, and α- and *β*-caseins was conducted.

### Patient characteristics and acquisition of milk samples

The University Maternity Hospital Limerick (UMHL) is the fifth largest maternity hospital in Ireland, with an in-house birth rate of 4,200 annually, and all preterm infants from 23 weeks of gestation are cared for locally.^22^ Resulting from a successful quality improvement project, 100% of ELBW infants were exposed to HM (MOM or DHM) consistently for the past 8 years contributing to one of the lowest NEC rates in Ireland.^23,24^ CMDF was used to fortify HM for ELBW infants until 2018, and from early 2019 all ELBW infants have received HMDF, thus offering an EHMD. UMHL reported 3 cases of NEC to Vermont Oxford Network among the 49 ELBW infants managed locally (total live births 18,018) during the pre-HMDF 4-year period, and 1 case of NEC during the post-HMDF introduction for equivalent duration (total ELBW 44, total live births 16,459). The single NEC reported during the post-HMDF period for ELBW was for a 26-week gestation infant and the event occurred post-transition to CMDF at 34 weeks, and not while in receipt of HMDF during the most vulnerable early gestational ages.

Following standardized procedures, FreMOM and FroMOM samples were procured in the morning (7–9 am), afternoon (1–3 pm), and night (7–9 pm), and were analyzed in triplicate to adjust for the diurnal variation. Neither maternal dietary restrictions, nor stipulation concerning the type of breast pump/suction pressure used to express BM at home were applied. For uniformity, hospital-grade electric pumps were used for sample acquisition at our lactation center (LC), and 1:1 mixed samples (mixture of foremilk and hindmilk) were used for analysis.

All MOM, PTDHM, and FTDHM samples were drawn from the surplus or discarded excess after feeding attempts, thus limiting wastage of invaluable HM. Individual analysis required only 0.5 to 2 mL per run. Single human milk bank (HMB) (Western Health & Social Care Trust, Enniskillen, Northern Ireland) supplied DHM during the study period. PTDHM was collected from mothers who gave birth <37 weeks of gestation and FTDHM from those >37 weeks. DHM specimens were kept frozen in transit from HMB to UMHL following the cold chain maintenance guidelines suggested by European Milk Bank Association (EMBA) and kept frozen at −20°C in a secure milk freezer in NICU, avoiding the chance of contamination.^7^ Frozen specimens were thawed and warmed to 37°C before feeding, using specified warmers (Beldico, Rue Andre Feher, Marche-en-Famenne, Belgium). HMB in Northern Ireland followed the recommended Holder pasteurization process (62.5°C or 144.5°F for 30 minutes) (low-temperature long-time) based on the EMBA guidelines.^7^

FreMOM specimens were transported from UMHL to the Bernal Institute laboratory, University of Limerick (5.7 km from NICU with 15 minutes' drive time, thus maintaining sample stability and recommended transport temperature), within 2 hours of collection during the daytime, and night samples were kept at 4°C until transport to laboratory in the morning. While all FroMOM, PTDHM, and FTDHM were kept at the same temperature (−20°C) at our LC, the latter two were postpasteurization, and MOM is frozen without undergoing pasteurization. All frozen samples used in the analysis were within 4 weeks of collection.

Bovine origin CMDF used in the analyses were preprepared, powdered, ready-to-go sachets kept at room temperature to be added to fresh or thawed HM as per the manufacturer's recommendations. CMDF prepared by two leading Irish manufacturers was used for the analysis, SMA^®^ (two sachets of 5 g each to 100 mL of HM) and C&G^®^ (one sachet of 2 g to 25 mL of HM). HMDF used for the analysis was Humavant™ +6, prepared from pooled donor BM, transported frozen from California to Limerick, Ireland at −70°C on dry ice, and thawed before mixing with HM at a prescribed ratio of 15 mL of HMDF to 35 mL of HM. HMDF produced by Prolacta Bioscience^®^ (City of Industry, CA) was the only FDA-approved preparation available during the study in Ireland.

### Experimental design for the milk analysis

A total of 25 milk specimens were analyzed. To account for gestation and postnatal age-specific variations, we included three FreMOM samples from mothers of infants born at 24-, 26-, and 28-week gestational ages and collected between 7 and 10 days of postnatal age, and each one was analyzed with and without fortification with either CMDF or HMDF. Three paired FroMOM specimens from the same mothers were kept frozen at LC for variable periods (ranging from 2 to 4 weeks), and each one was analyzed with and without CMDF or HMDF fortification. Two DHM specimens (one PTDHM and one FTDHM) were also analyzed with and without added CMDF or HMDF. Three commercial samples from preprepared bovine-based PTF and term formulas (two PTF and one TF) were analyzed as comparators. The resultant 25 milk specimens are labeled in Table 1.

**Table 1.** Specimen Codes for Milk Specimen Analysis

PTDHM	Preterm donor human milk (banked milk)
PTDHM+CMDF	Preterm donor human milk+cow milk-derived multinutrient fortifier
PTDHM+HMDF	Preterm donor human milk+human milk-derived fortifier
FTDHM	Term donor human milk (banked milk)
FTDHM+CMDF	Term donor human milk+cow milk-derived multinutrient fortifier
FTDHM+HMDF	Term donor human milk+human milk-derived fortifier
FreMOM – 24 weeks	Fresh milk sample from mother of infant born at 24 weeks gestational age
FreMOM – 26 weeks	Fresh milk sample from mother of infant born at 26 weeks gestational age
FreMOM – 26 weeks+CMDF	Fresh milk sample from mother of infant born at 26 weeks+cow milk-derived multinutrient fortifier
FreMOM – 26 weeks+HMDF	Fresh milk sample from mother of infant born at 26 weeks+human milk-derived fortifier
FreMOM – 28 weeks	Fresh milk sample from mother of infant born at 28 weeks gestational age
FreMOM – 28 weeks+CMDF	Fresh milk sample from mother of infant born at 28 weeks+cow milk-derived multinutrient fortifier
FreMOM – 28 weeks+HMDF	Fresh milk sample from mother of infant born at 28 weeks+human milk-derived fortifier
FroMOM – 24 weeks	Frozen milk sample from same mother of infant born at 24 weeks gestational age
FroMOM – 24 weeks+CMDF	Frozen milk sample from same mother of infant born at 24 weeks gestational age+cow milk-derived multinutrient fortifier
FroMOM – 24 weeks+HMDF	Frozen milk sample from same mother of infant born at 24 weeks gestational age+human milk-derived fortifier
FroMOM – 26 weeks	Frozen milk sample from same mother of infant born at 26 weeks gestational age
FroMOM – 26 weeks+CMDF	Frozen milk sample from same mother of infant born at 26 weeks gestational age+cow milk-derived multinutrient fortifier
FroMOM – 26 weeks+HMDF	Frozen milk sample from same mother of infant born at 26 weeks gestational age+human milk-derived fortifier
FroMOM – 28 weeks	Frozen milk sample from same mother of infant born at 28 weeks gestational age
FroMOM – 28 weeks+CMDF	Frozen milk sample from same mother of infant born at 28 weeks gestational age+cow milk-derived multinutrient fortifier
FroMOM – 28 weeks+HMDF	Frozen milk sample from same mother of infant born at 28 weeks gestational age+human milk-derived fortifier
CBPTF-1	Commercial preprepared bovine-based preterm formula (company 1)
CBPTF-2	Commercial preprepared bovine-based preterm formula (company 1) specific for low-birthweight baby
CBTF	Commercial preprepared bovine-based term formula (company 2)

CMDF, cow's milk-derived fortifier; FreMOM, freshly expressed mother's own milk; FroMOM, frozen mother's own milk; FTDHM, full-term donor human milk; HMDF, human milk-derived fortifier; PTDHM, preterm donor human milk.

A detailed description of biochemical and immunochemical analyses is given as Supplementary Data (Appendix A1).

### Patient and public involvement

Irish neonatal health alliance (INHA), a patient advocacy group (Charity registration number: 20100100) representing parents of premature infants, was involved from the conception to completion of the study. INHA reviewed the study design, assisted in the design of patient-facing materials, and reaffirmed the research relevance to preterm infants and families. Participation of Western Trust Donor Milk Bank, our sole supplier of DHM as a collaborator, also endorses the Patient and Public Involvement (PPI) commitment.

### Research Ethics Committee approval

The University of Limerick Hospital Group Research Ethics Committee (REC) approved the study (No: 10/21). Fully anonymized data were used, and Irish Health Service Executive directives on general data protection regulation guidelines were followed.^25^ Written informed consent was obtained from each enrolled mother who offered FreMOM and FroMOM specimens. Consent was also obtained (through the Western Trust Donor Milk Bank) from donors who supplied the DHM.

### Statistical analysis

Analysis of variance was performed using Minitab^®^ 18.1 (MINITAB, Inc., Coventry, United Kingdom), applying the general linear model procedure and Tukey's test for pairwise comparison. All the measurements were carried out in triplicate, and the results were expressed as the mean value ±standard deviation.

## Results

### Chemical and physicochemical composition

The pH values of specimens ranged from 6.38 to 6.87, and freezing showed no significant effect (*p* > 0.05) on the human milk pH values (Table 2). The fat content showed significant differences (*p* < 0.05) between HM samples with a lower range (2.39% to 2.6%) for FreMOM (24 and 26 weeks), FroMOM (28 weeks), and CBTF. HMDF-added specimens exhibited significantly higher protein and total solids (*p* < 0.05) than unfortified samples and, to some extent, with the CMDF-fortified samples, reflecting the contribution of HMDF in increasing macronutrients. HMDF fortification has also significantly (*p* < 0.05) increased the fat content compared with unfortified counterparts (Table 2). Moreover, freezing (FroMOM) showed no significant (*p* > 0.05) lowering of fat, protein, and total solid values (Table 2).

**Table 2.** Chemical Composition of 25 Human Milk Samples (22 Human Milk and 3 Formula Specimens)

	pH value	Fat (%)	Protein (%)	Total solids (%)	Antioxidant activity (%)
PTDHM	6.51 ± 0.03^gh^	3.80 ± 0.10^fg^	2.05 ± 0.13^h^	12.46 ± 0.14^k^	42.40 ± 1.73^jk^
PTDHM+CMDF	6.42 ± 0.01^h^	3.67 ± 0.06^g^	3.16 ± 0.05^fg^	15.76 ± 0.09^e^	55.77 ± 1.95^gh^
PTDHM+HMDF	6.43 ± 0.01^h^	5.27 ± 0.06^b^	3.49 ± 0.03^de^	15.97 ± 0.12^de^	67.66 ± 1.28^c^
FTDHM	6.61 ± 0.03^fg^	4.20 ± 0.10^de^	2.30 ± 0.10^h^	12.43 ± 0.20^k^	54.61 ± 1.54^gh^
FTDHM+CMDF	6.38 ± 0.04^h^	4.03 ± 0.06^ef^	3.20 ± 0.15^f^	16.08 ± 0.15^cde^	63.28 ± 1.43^cde^
FTDHM+HMDF	6.44 ± 0.04^h^	5.87 ± 0.06^a^	3.54 ± 0.18^de^	16.31 ± 0.10^bcd^	79.08 ± 1.28^b^
FreMOM – 24 weeks	6.83 ± 0.05^ab^	2.60 ± 0.10^lm^	2.10 ± 0.15^h^	11.28 ± 0.11^m^	26.90 ± 1.7^m^
FreMOM – 26 weeks	6.80 ± 0.04^abcde^	2.83 ± 0.06^l^	2.24 ± 0.19^h^	11.75 ± 0.09^l^	53.76 ± 1.48^h^
FreMOM – 26 weeks+CMDF	6.61 ± 0.03^fg^	2.60 ± 0.10^lm^	3.20 ± 0.16^f^	14.77 ± 0.09^fg^	58.74 ± 1.23^efg^
FreMOM – 26 weeks+HMDF	6.65 ± 0.05^ef^	4.01 ± 0.09^ef^	3.37 ± 0.17^def^	14.89 ± 0.10^f^	63.07 ± 1.54^cde^
FreMOM – 28 weeks	6.81 ± 0.04^abcd^	3.40 ± 0.10^h^	3.16 ± 0.19^fg^	13.78 ± 0.13^i^	62.92 ± 1.19^cde^
FreMOM – 28 weeks+CMDF	6.67 ± 0.05^cdef^	3.23 ± 0.06^ij^	4.04 ± 0.19^c^	16.38 ± 0.14^bc^	74.64 ± 1.15^b^
FreMOM – 28 weeks+HMDF	6.64 ± 0.03^fg^	4.80 ± 0.10^c^	4.25 ± 0.16^abc^	16.61 ± 0.10^ab^	79.34 ± 1.44^b^
FroMOM – 24 weeks	6.87 ± 0.03^a^	3.23 ± 0.15^ig^	3.18 ± 0.19^fg^	11.33 ± 0.15^m^	26.38 ± 1.51^m^
FroMOM – 24 weeks+CMDF	6.86 ± 0.05^ab^	3.04 ± 0.10^k^	4.15 ± 0.08^bc^	14.94 ± 0.12^f^	42.62 ± 1.64^j^
FroMOM – 24 weeks+HMDF	6.74 ± 0.07^abcde^	4.41 ± 0.08^d^	4.39 ± 0.11^ab^	15.17 ± 0.13^f^	49.14 ± 1.75^i^
FroMOM – 26 weeks	6.81 ± 0.07^ab^	4.37 ± 0.06^d^	3.30 ± 0.05^ef^	13.10 ± 0.09^j^	57.43 ± 1.19^fgh^
FroMOM – 26 weeks+CMDF	6.66 ± 0.06d^ef^	4.20 ± 0.10^de^	4.12 ± 0.08^bc^	16.03 ± 0.12^cde^	64.53 ± 1.51^cd^
FroMOM – 26 weeks+HMDF	6.66 ± 0.04^ef^	5.96 ± 0.13^a^	4.50 ± 0.10^a^	16.81 ± 0.12^a^	75.64 ± 1.34^b^
FroMOM – 28 weeks	6.87 ± 0.05^a^	2.50 ± 0.10^mn^	2.21 ± 0.11^h^	12.23 ± 0.14^k^	37.74 ± 1.27^kl^
FroMOM – 28 weeks+CMDF	6.74 ± 0.06^abcde^	2.30 ± 0.08^n^	3.35 ± 0.10^def^	14.48 ± 0.16^gh^	57.65 ± 1.45^fgh^
FroMOM – 28 weeks+HMDF	6.81 ± 0.04^abc^	3.62 ± 0.13^g^	3.52 ± 0.12^de^	14.91 ± 0.19^f^	61.15 ± 1.36^def^
CBPTF-1	6.74 ± 0.07^abcde^	3.81 ± 0.11^fg^	3.61 ± 0.12^d^	16.85 ± 0.11^a^	84.87 ± 1.92^a^
CBPTF-2	6.82 ± 0.08^ab^	3.30 ± 0.10^ij^	3.14 ± 0.09^fg^	15.75 ± 0.07^e^	49.15 ± 2.05^i^
CBTF	6.72 ± 0.05^bcdef^	2.39 ± 0.07^mn^	2.92 ± 0.13^g^	14.24 ± 0.14^h^	34.87 ± 1.74^l^

Data are mean ± standard deviation (*n* = 3); values in the same column with different superscript letters are significantly different (*p* < 0.05).

CMDF, cow's milk-derived fortifier; FreMOM, freshly expressed mother's own milk; FroMOM, frozen mother's own milk; FTDHM, full-term donor human milk; HMDF, human milk-derived fortifier; PTDHM, preterm donor human milk.

### Antioxidant activity

**FIG. 1.**
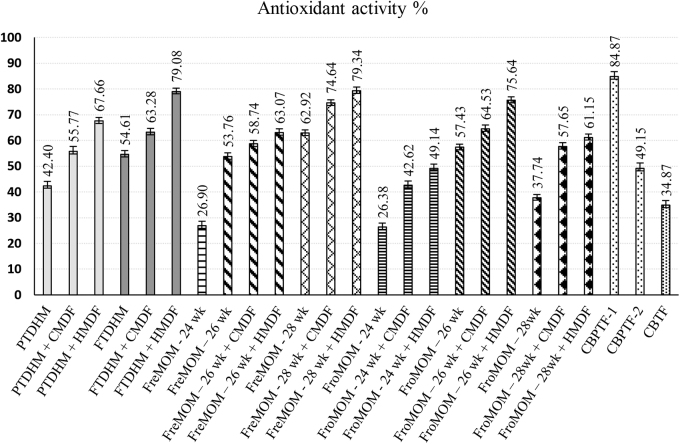
Antioxidant activity of DHM, MOM (fresh and frozen) with HMDF, and CMDF supplementation and commercial preprepared bovine-based formulas expressed as percentage. Values are mean of triplicate measurements with their standard deviations represented by vertical bars. AA, antioxidant activity; CMDF, cow's milk-derived fortifier; DHM, donor human milk; HMDF, human milk-derived fortifier; MOM, mothers' own milk.

AA determined using the 2,2-diphenyl-1-picryl-hydrazyl-hydrate (DPPH) method is summarized in Table 2 and Figure 1. Among HM specimens, HMDF-fortified samples showed significant (*p* < 0.05) and the highest AA, followed by CMDF-fortified samples compared with the corresponding unfortified specimens. HMDF addition increased the percentage of AA by almost twice that of unfortified specimens, particularly with those samples of lower AA (FreMOM 24 weeks and FroMOM 28 weeks), underpinning the capability of HMDF to increase oxidative scavenging and protecting premature infant gut against oxidative stress and free radical influences. Significant (*p* < 0.05) variations have been noted among HM specimens of different gestations (24, 26, and 28 weeks) with the lowest AA reported for the 24-week gestation sample (26.38% and 26.90%). Compared with FreMOM, AA was lower in frozen and pasteurized samples. Significant variation has also been noted within the three commercially preprepared formulas, where CBPTF-1 has the highest (84.87%) and CBTF the lowest AA (34.87%).

### Reversed-phase high-performance liquid chromatography data

**FIG. 2.**
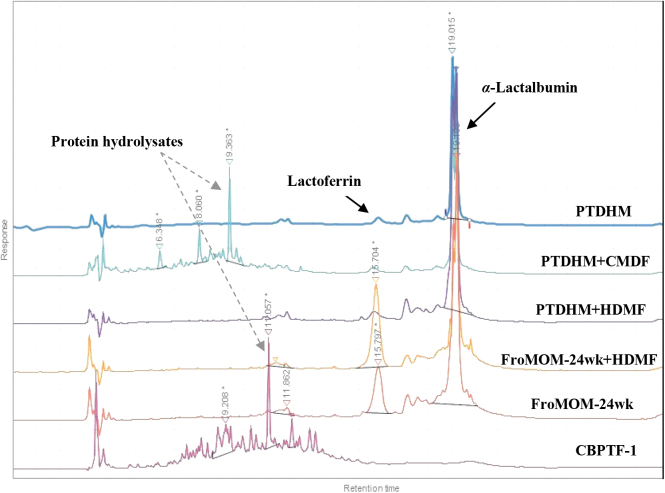
Reversed-phase HPLC of six milk specimens: PTDHM, PTDHM fortified with CMDF, PTDHM fortified with HMDF, FroMOM-24 weeks fortified with HMDF, FroMOM-24 weeks without fortification and CBPTF-1. CBPTF-1, commercial preprepared term type; CMDF, cow's milk-derived fortifier; FroMOM, frozen mother's own milk; PTDHM, preterm donor human milk; HMDF, human milk-derived fortifier; HPLC, high-performance liquid chromatography.

To visualize variations in major human milk proteins, chromatograms derived from six specimens representing various samples were overlaid (Fig. 2). Commercial preprepared samples (CBPTF-1, CBPTF-2, and CBTF) and all HM specimens fortified with CMDF showed various new peptides in the hydrophilic region, most probably protein hydrolysates. In addition, a complete absence of α-LA retention time (RT): 19 ± 0.5 minutes and LF RT: 15 ± 0.5 minutes was noted with bovine-based formula, except that a barely perceptible α-LA was observed on one CBTF specimen. HM samples fortified with HMDF showed significantly higher α-LA content (*p* < 0.05), whereas the addition of CMDF decreased the net α-LA, suggesting that CMDF fortifier is devoid of α-LA. This observation was confirmed by the absence of α-LA peak in the pure CMDF chromatogram (data not shown), as well as the sodium dodecyl sulfate–polyacrylamide gel electrophoresis (SDS-PAGE) results that will be discussed later.

**FIG. 3.**
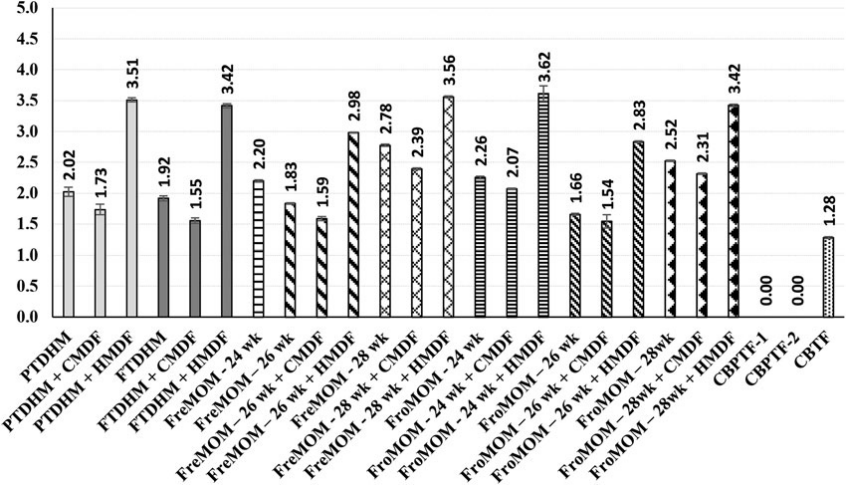
Alpha-lactalbumin content (mg/mL) of DHM and MOM (fresh and frozen) supplemented with HMDF and CMDF and commercial preprepared bovine-based formulas. Values are means of triplicate measurements with their standard deviations represented by vertical bars. Column headed by α-LA content. CMDF, cow's milk-derived fortifier; DHM, donor human milk; HMDF, human milk-derived fortifier; α-LA, α-lactalbumin; MOM, mothers' own milk.

It is worth noting that the concentration of α-LA in DHM (PTDHM and FTDHM, Fig. 3) showed comparable values to HM specimens from mothers of infants born at 24- and 26-week gestational ages (both FreMOM and FroMOM). For the bovine-based formula, only one sample of CBTF exhibited a noticeable concentration of α-LA (1.28 mg/mL). Nevertheless, it was significantly low (*p* < 0.05) compared with HM specimens (Fig. 3).

**FIG. 4.**
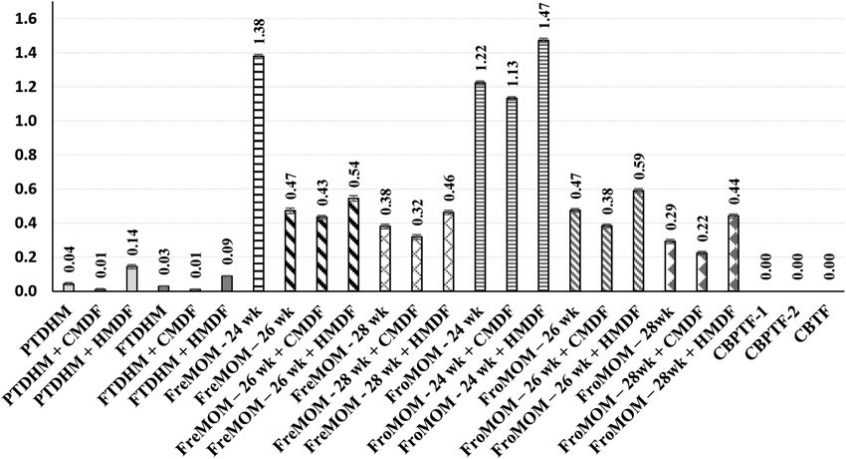
Lactoferrin content (mg/mL) of DHM and MOM (fresh and frozen) supplemented with HMDF and CMDF and commercial preprepared bovine-based formulas. Values are means of triplicate measurements with their standard deviations represented by vertical bars. Column headed by Human LF content. CMDF, cow's milk-derived fortifier; DHM, donor human milk; HMDF, human milk-derived fortifier; LF, Lactoferrin; MOM, mothers' own milk.

For LF, the addition of HMDF showed a similar trend as obtained for α-LA, significantly higher content (*p* < 0.05) compared with nonfortified counterparts (Fig. 4). However, DHM per se exhibited significantly low (*p* < 0.05) LF concentrations and were barely perceptible (0.03 and 0.04 mg/mL). None of the bovine-based formulae showed any identifiable amounts of LF (Fig. 3). MOM of infants born at 24 weeks of gestational age (both fresh and frozen) exhibited the highest LF content (1.38 and 1.22 mg/mL), and values significantly decreased (*p* < 0.05) with increasing gestational age (Fig. 4).

### Electrophoresis

**FIG. 5.**
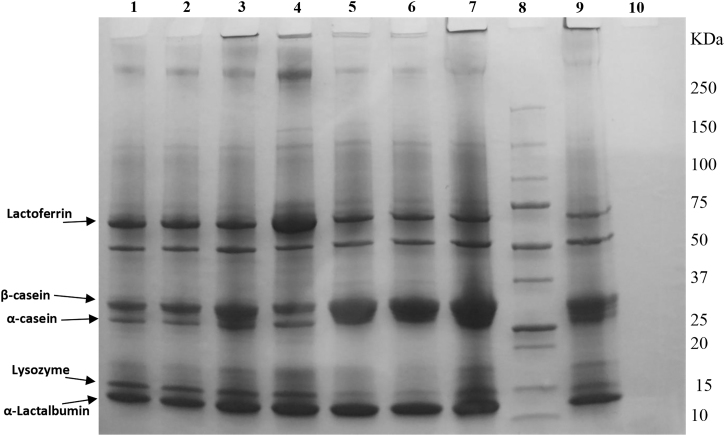
Reducing SDS-PAGE patterns of seven fresh human milk samples “FreMOM” of mothers of infants born at 24, 26, and 28-week gestational ages with or without fortifications. Lane1: FreMOM – 24 weeks. Lane 2: FreMOM – 26 weeks. Lane 3: FreMOM – 26 weeks+CMDF. Lane 4: FreMOM – 26 weeks+HMDF. Lane 5: FreMOM – 28 weeks. Lane 6: FreMOM – 28 weeks+CMDF. Lane 7: FreMOM – 28 weeks+HMDF. Lane 9: Pure human milk-based fortifier “HMDF.” Lane 10: pure bovine-based powder fortifier “CMDF.” Molecular mass markers (10–250 kDa) are in Lane 8. CMDF, cow's milk-derived fortifier; FreMOM, freshly expressed mother's own milk; HMDF, human milk-derived fortifier; SDS-PAGE, sodium dodecyl sulfate–polyacrylamide gel electrophoresis.

HMDF addition noticeably increased content of most of the bioactive HM proteins, particularly α-LA, lysozyme, and LF (Fig. 5). The α- and *β*-caseins, demonstrated by the higher intensities of protein bands in lanes 4 and 7, also increased through HMDF fortification compared with the unfortified specimens. This result could be explained by the protein profiles of the pure HMDF and CMDF fortifiers presented in lanes 9 and 10, respectively, where a complete absence of major human milk bioactive proteins (LF, α-LA, lysozyme, and α- and *β*-caseins) was noted with pure CMDF (Lane 10), an observation that goes parallel with the previously discussed high-performance liquid chromatography (HPLC) data.

**FIG. 6.**
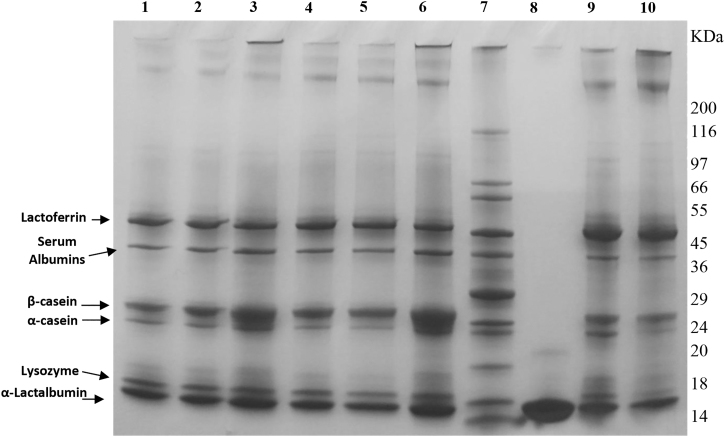
Reducing SDS-PAGE patterns of fresh and frozen human milk samples of mothers of infants born at 24 and 26-week gestational ages with or without fortifications. Lane1: FreMOM–26 weeks. Lane 2: FreMOM–26 weeks+CMDF. Lane 3: FreMOM–26 weeks+HMDF. Lane 4: FroMOM–26 weeks. Lane 5: FroMOM–26 weeks+CMDF. Lane 6: FroMOM–26 weeks+HMDF. Lane 8: Alpha-Lactalbumin human milk standard. Lane 9: FreMOM–24 weeks. Lane 10: FroMOM–24 weeks. Molecular mass markers (6.5–200 kDa) are in Lane 7. CMDF, cow's milk-derived fortifier; FreMOM, freshly expressed mother's own milk; HMDF, human milk-derived fortifier; SDS-PAGE, sodium dodecyl sulfate–polyacrylamide gel electrophoresis.

The sodium dodecyl sulfate (SDS) protein patterns presented in Figure 6 reveal a considerable amount of major milk proteins, indicated by the intensity rate of protein bands, present in both preterm and term DHM (Lanes 1–6). However, lysozyme could only be noted in our study with MOM (both fresh and frozen) and the HMDF samples (Figs. 5 and 6). Among the three bovine-based commercial samples, only the term formula (CBTF) showed even low-intensity bands of α-LA, and α- and *β*-caseins, whereas complete absence of any bioactive protein bands was noted with both the preterm formula (CBPTF-1 and CBPTF-2; data not shown). These results are consistent with HPLC data (Figs. 2–4) and demonstrate the variety of bioactive proteins in HM specimens and potential of HMDF fortification to augment the concentrations for the ELBW population.

Overall, FreMOM and FroMOM specimens fortified with HMDF exhibited the highest levels of measured bioactive proteins, whereas CMDF addition decreased their final net concentration. Nevertheless, CMDF improved the AA to some extent, attributable to its protein hydrolysate content, but still significantly lower than yielded through HMDF fortification. While offering multinutrient fortification, bovine origin CMDF per se did not add measurable bioactive protein values among tested specimens.

## Discussion

In addition to the core nutritional role, HM, through its immunological influence, fends off infections, limits intestinal inflammatory changes, and improves the overall survival of preterm infants. The advent of HM fortification enabled ELBW and VLBW infants to optimize tissue accretion and prevent extrauterine growth restriction.^7,9^ However, the traditional CMDF lacks bioactive and immune components that protect vulnerable VPT infants. Moreover, cow's milk protein, as present in CMDF, has been established to increase the incidence of NEC.^5,12,16,21^

This study represents one of the first analyses of HM at different gestations in its fresh, frozen, and banked forms, with the paired fortification of CMDF and HMDF suggesting a distinct benefit of the latter. The abundance of biologically active proteins and immune mediators in colostrum and fresh maternal BM makes it “nature's first vaccine,” especially for the VPT and VLBW infants.^26^ They are largely devoid of the transplacental transfer of maternal IgG antibodies, which occurs in the last trimester.^26,27^ Variations in bioactive proteins in HM could be influenced by maternal factors (gestational age, lactational stage, maternal nutrition, and body mass index), collection factors (foremilk versus hindmilk, type of breast pump—manual or mechanical, timing of pumping, diurnal–nocturnal influences) as well as processing factors (freezing, method of pasteurization, immersion techniques, heat treatments, and thawing options).^28–30^

HM shows wide variations in protein, fat, and carbohydrate contents, with DHM having a significantly lower concentration of all of the above.^31^ Compared with CMDF, in our analysis, HMDF yielded significantly enhanced measurable amount of fat (2.3–2.8% versus 4.4–6.1%) and modestly increased total protein 3.16–4.15% versus 3.49–4.5% (Table 2).

AA yielded consistently high measurements for all HMDF-added specimens. Hanson et al. reported an 18–53% reduction of AA of DHM compared with MOM.^32^ Our study resonates the same and additionally demonstrates HMDF augmenting the antioxidant property of DHM. AA of HM is crucial in reducing oxidative damage to developing tissues.^33^ Although unexpected, one of the samples of PTF in our study showed relatively high AA. The commonest whey protein in HM is α-LA, accounting for 20–25% of total HM proteins, whereas it contributes only to 3.5% in bovine origin milk.^34^ The predominant protein in bovine milk, *β*-lactoglobulin is not present in HM.^34^ α-LA in HM offers varied and unique biological properties through the following: (1) role in lactose formation (lactase synthase pathway) in mammary epithelium; (2) presence of bioactive peptides and essential amino acids such as tryptophan, lysine, isoleucine, and valine; (3) modulation of the immunological and cellular functions of the developing gut; (4) regulation of sleep/wake cycles in the infant; and (5) prebiotic, antibacterial, and anticancer properties.^17,34^

LF, the second main whey protein fraction is one of the most abundant glycoproteins in HM. With the highest concentration in colostrum and preterm fresh MOM, LF is a key immunoprotective protein having a nonspecific defense against microbial and viral infections and with its iron-binding property selectively binds to LF receptors in the small intestine, monocytes, and lymphocytes.^32,35^ Lysozyme is part of the innate immune defense and causes the lysis of pathogenic bacterial cell walls while allowing protective bifidobacterial growth.^30^ Secretory IgA has already been fairly well studied in HM and was not measured in our analysis.^32^ Similarly, leptin is the most resistant to freezing, heating, and pasteurization and thus was not further analyzed.^30^ Our results of low levels of LF, α-LA, and lysozyme in the banked DHM, reflecting some previous reports, warrant further exploration of the potential reasons, plausible implications, and possible solutions. Suggested additional benefits of LF, α-LA, and lysozyme include immunomodulatory, trophic, anti-infective, antioxidant, anti-inflammatory, iron chelation, bactericidal, antiadhesive, and antiobesity properties.^30,32–35^

HM contains β-casein and k-casein while being devoid of α-casein (bovine origin).^17,18^ β-Casein is the most detectable bioactive fragment among the HM peptides.^36^ As with previous studies, despite freezing and pasteurization, identifiable levels of certain bioactive peptides were noted in our DHM specimens.^36^ HMDF used in our analysis was processed using Vat pasteurization (Vat-PT) (also known as batch or jacket pasteurization), as per the manufacturer's specifications. The choice of pasteurization (Vat-PT, retort sterilization [RTR], or ultra-high-temperature [UHT] processing) as well as thawing and homogenization could influence the structure of bioactive proteins in DHM.^37^ Overall, Vat-PT preserves more of bioactive proteins compared with UHT or RTR.^37^ Similarly, traditional Holder pasteurization of DHM, while retaining the macronutrient concentrations, significantly reduces the bioactivity, including considerable loss of lysozyme and lactoferrin.^38^ Comparable to our observation, Akinbi et al. and Chang et al. reported preservation of bioactive protein in FroMOM compared with pasteurized DHM.^19,39^

Chang et al. also noticed preservation of lysozyme compared with other bioactive proteins during the freezing of MOM (without undergoing pasteurization).^39^ One recent nonsystematic review summarized the reduction of various bioactive components through DHM pasteurization (LF reduction by 44% to 91%, LZ by 59%, antioxidant capacity by 67%, and secretory IgA by 51%).^31^ Enhancement of LF, α-LA, and LZ levels with the addition of HMDF (*and not with CMDF*) to PTDHM, FTDHM, and MOM, as demonstrated in this study, would favor such a nutritional choice. Our corroborating clinical observation of a very low NEC rate at UMHL that maintains 100% HM exposure (65% being FreMOM), along with HMDF to ELBW infants would also support the potential clinical relevance of bioactive properties of HM.^23,24^

Individualized fortification (either as targeted or adjustable) of each MOM feed offered to ELBW infants would be ideal and has been encouraged by EMBA.^7^ However, the logistical and personnel limitations curtail the widespread and routine clinical application.^40^ Recent studies aimed at evaluating the role of MOM and EHMD in modifying gut microbial colonization, and later vascular aging and neurodevelopmental outcomes could possibly reaffirm the uniqueness of HM bioactivity.^5,41,42^ Our study contributes to new information that specific bioactive properties lost or attenuated through the pasteurization of DHM could be reinstated through HMDF, and for MOM, the levels could be augmented offering potential benefits to ELBW infants. An improved understanding of how MOM designs, donates, and modulates the immunochemical components to offspring would be desirable to improve preventative approaches.

### Limitations

The following limitations are acknowledged: (1) The relatively small number of gestation and age-specific specimens, (2) Maternal characteristics (including ethnic and dietetic) potentially influencing the composition of MOM were not controlled, (3) Precise gestation and collection time of the individual DHM donations were not ascertained, (4) Qualitative or in-vivo bioactivity measurements were not conducted, (5) DHM undergoing newer pasteurization techniques or samples kept frozen for extended periods were not analyzed, (6) The same MOM specimen could not be paired to yield frozen and pasteurized samples, (7) The list of bioactive proteins studied is not exhaustive, and (8) Human milk oligosaccharides, free fatty acids, and milk fat globules were not analyzed. However, given the spectrum of triads of specimens studied (fresh, frozen, pasteurized, and each with dual fortification), our unique results support the value of HMDF in reinstating and enhancing bioactive components in HM.

## Conclusion

Pasteurized DHM offers significantly low bioactive properties compared with fresh and frozen MOM. In contrast to CMDF, HMDF contributes additional bioactive components to ELBW infants. With multiple reports suggesting attenuation of bioactivity of DHM through pasteurization, our analysis demonstrates a novel method to reinstate bioactive proteins by adding HMDF. Frozen MOM retained better quantitative bioactivity compared with banked pasteurized DHM. Based on our observations, we recommend freshly expressed MOM fortified with HMDF as an EHMD, offering bioactive programming for very premature infants during the early critical postnatal window (*3E—early*, *enterally, exclusively*). Further research in personalized enteral nutrition, gut microbiome, organoids, and artificial intestinal models, could shed light on the precise mechanisms through which MOM exerts the unique immunological imprinting to her newborn infant.

## Data Availability

All data relevant to the study are included in the article and details of laboratory methodology provided as Appendix A1.

## Patient and Public Involvement

Through the registered charity and parent organization, PPI was ensured in the design, REC submission, article development, and authorship.

## Appendix A1.

### Biochemical and Immunochemical Analyses

#### Materials and chemicals

HPLC-grade acetonitrile, trifluoroacetic acid (TFA), and methanol were purchased from Sigma-Aldrich (Dublin, Ireland). DPPH was obtained from Thermo Fisher Scientific (Dublin). LA from human milk (HLA, ≥95% purity, lyophilized powdered) LF from human milk (HLF, ≥85% purity, lyophilized powder) standards (Sigma-Aldrich) were used. All other reagents and chemicals were of analytical grade.

#### Chemical composition

The total nitrogen content was measured by the Kjeldahl method (AOAC, 2000) and then multiplied by the conversion factor 6.25 for human milk protein to calculate the total protein in human milk samples.^A1,A2^ The total solid and fat contents of human milk were determined by a SMART-6™ Analyzer and ORACLE Universal Fat Analyzer, respectively (CEM Corporation, NC). The pH values were measured using a digital pH-meter (InoLab^®^ 7110; WTW, Weilheim, Germany).

#### Antioxidant activity

Radical scavenging assay of DPPH was used to evaluate the AA of the human milk samples according to Abdel-Hamid et al.^A3,A4^ Briefly, 3 mL diluted human milk sample (1:10 using MilliQ-water) was mixed with 3 mL freshly prepared DPPH reagent (0.2 mL, dissolved in 95% methanol) and then incubated in dark at 37°C or 98.6°F for 30 minutes. The absorbance was measured at 517 nm.

#### SDS-PAGE electrophoresis

Evaluation of the milk proteins and comparison between the different HM specimens and commercial infant formulas was performed by SDS-PAGE technique under reducing conditions.^A5^ Mini-PROTEAN tetra system (Bio-Rad Laboratories, Inc.) and mini-PROTEAN TGX^®^ stain-free precast gels (4–20%; Bio-Rad Laboratories, Inc.) were used. The gels were stained with 0.25% (w/v) Coomassie-R in 10% (v/v) acetic acid and 40% (v/v) methanol. Protein bands were determined according to their molecular weight by comparison with a molecular weight marker (6.5–200 or 10–250 kDa; Sigma-Aldrich).

#### Reversed phase-HPLC

The major proteins of human milk, LA, and LF, were determined using RP-HPLC (Agilent 1260 Infinity Series, Waldbronn, Germany) according to the method described by Yuksel and Erdem in 2010 with some modification.^A6^ Briefly, 2 mL of milk sample was defatted by centrifugation at 3,000 *g* at 4°C for 15 minutes. The skimmed milk was transferred to a new tube without retrieving the cream layer and diluted with MilliQ-water; to preserve the natural status of the milk proteins, to reach 0.5% (v/v) milk protein concentration. Before injection, the milk aliquots were filtered through a 0.45 μm cellulose acetate syringe filter (Millex^®^ GP; Merck Millipore Ltd., Ireland). The Agilent 1260 RP-HPLC system consisted of quaternary pump (Agilent G1311B), autosampler (Agilent G1329B), vacuum degasser (Agilent G1379A), temperature-controlled column compartment (Agilent G1316A), and DAD VL detector (Agilent G1315D).^A7^ The column used was Agilent Zorbax column (300SB-C18, 4.6 × 250 mm, 5 μm particle size), and its temperature was maintained at 25°C during protein separation.^A7^ The eluents were solvent A: TFA and MilliQ-water in a ratio of 1:1,000 (v/v), and solvent B: TFA, acetonitrile, and MilliQ-water in a ratio of 1:900:100 (v/v/v). The gradient elution profile was 0–5 minutes: 10–25% B; 5–12 minutes: 25–42% B; 12–17 minutes: 42–75% B; 17–19 minutes: 75–90% B; 19–24 minutes: 90% B, 24–26 minutes: 90–95% B, then at 26–30 minutes: 5% B. Twenty microliter milk aliquots was injected and eluted at a flow rate of 0.8 mL/min and the eluted proteins were detected at 215 nm.

MilliQ-water was used to prepare a final standard stock solution concentration of 5 mg/mL of HLA and HLF. A series of six dilutions of each standard stock solution were prepared using MilliQ-water. A standard curve was obtained by plotting the peak area against six concentrations of the standard solutions in the range of 0.156–5 mg/mL with *R*-squared (*R*^2^) for the linearity of the curves of 0.995 and 0.992 for HLA and HLF, respectively. Data were collected and processed by Agilent OpenLab 2.2 chromatography data systems software. The LA and LF concentrations of the milk samples were determined through the total area under the curve of the chromatogram peak and the standard curves.
